# Comparison of Cardioprotective Potential of Cannabidiol and β-Adrenergic Stimulation Against Hypoxia/Reoxygenation Injury in Rat Atria and Ventricular Papillary Muscles

**DOI:** 10.3390/ph17101379

**Published:** 2024-10-16

**Authors:** Anna Pędzińska-Betiuk, Ulrich Gergs, Jolanta Weresa, Patryk Remiszewski, Ewa Harasim-Symbor, Barbara Malinowska

**Affiliations:** 1 Department of Experimental Physiology and Pathophysiology, Medical University of Bialystok, 15-222 Bialystok, Poland; jolanta.weresa@umb.edu.pl (J.W.); patryk.remiszewski@umb.edu.pl (P.R.); barbara.malinowska@umb.edu.pl (B.M.); 2 Institute for Pharmacology and Toxicology, Medical Faculty, Martin Luther University Halle-Wittenberg, 06097 Halle, Germany; ulrich.gergs@medizin.uni-halle.de; 3 Department of Physiology, Medical University of Bialystok, 15-222 Bialystok, Poland; ewa.harasim-symbor@umb.edu.pl

**Keywords:** hypoxia/reoxygenation, isolated atria, isolated ventricular papillary muscles, cannabidiol, isoprenaline, hypertension

## Abstract

Background: Hypoxia is one of the most significant pathogenic factors in cardiovascular diseases. Preclinical studies suggest that nonpsychoactive cannabidiol (CBD) and β-adrenoceptor stimulation might possess cardioprotective potential against ischemia-reperfusion injury. The current study evaluates the influence of hypoxia-reoxygenation (H/R) on the function of atria and ventricular papillary muscles in the presence of CBD and the nonselective β-adrenoceptor agonist isoprenaline (ISO). Methods: The concentration curves for ISO were constructed in the presence of CBD (1 µM) before or after H/R. In chronic experiments (CBD 10 mg/kg, 14 days), the left atria isolated from spontaneously hypertensive (SHR) and their normotensive control (WKY) rats were subjected to H/R following ISO administration. Results: Hypoxia decreased the rate and force of contractions in all compartments. The right atria were the most resistant to hypoxia regardless of prior β-adrenergic stimulation. Previous β-adrenergic stimulation improved recovery in isolated left atria and right (but not left) papillary muscles. Acute (but not chronic) CBD administration increased the effects of ISO in left atria and right (but not left) papillary muscles. Hypertension accelerates left atrial recovery during reoxygenation. Conclusions: H/R directly modifies the function of particular cardiac compartments in a manner dependent on cardiac region and β-adrenergic prestimulation. The moderate direct cardioprotective potential of CBD and β-adrenergic stimulation against H/R is dependent on the cardiac region, and it is less than in the whole heart with preserved coronary flow. In clinical terms, our research expands the existing knowledge about the impact of cannabidiol on cardiac ischemia, the world′s leading cause of death.

## 1. Introduction

The multitarget, nonpsychoactive cannabidiol (CBD) is one of the most promising molecules in the family of cannabinoids. Its beneficial properties and therapeutic significance result from its antioxidant, anti-inflammatory, immunomodulatory, antiproliferative, antiapoptotic, and neuroprotective activities [1,2]. Thus, CBD exhibits therapeutic potential in a wide number of diseases, including those affecting the central nervous system (e.g., epilepsy, multiple sclerosis, Parkinson’s disease, and Alzheimer’s disease), pain, and potentially cancer [3,4].

Additionally, the therapeutic potential of CBD is also suggested in cardiovascular diseases, such as diabetic cardiomyopathy, myocarditis, doxorubicin-induced cardiotoxicity, and myocardial ischemia-reperfusion injury [2,5,6,7]. Importantly, the American Food and Drug Administration (FDA) has recently approved the use of CBD for the treatment of seizures, and clinical trials are currently underway to test CBD′s efficacy in the treatment of myocarditis and pericarditis [2,8]. Moreover, medical cannabis with a high CBD content in cannabis oil, in conjunction with standard cardiac care, has been demonstrated in a case report to be responsible for the improved functional capacity of a 63-year-old patient with unstable angina [9].

Hypoxia is one of the most significant pathogenic factors in cardiovascular diseases [10]. It results in irreversible necrosis of tissue and cardiac failure. Reperfusion restores the blood supply to the heart, but paradoxically, it exacerbates and accelerates by itself the damage in the myocardium referred to as ischemia-reperfusion (I/R) injury [11]. Chronic CBD administration has been demonstrated in vivo and/or in perfused hearts to reduce infarct size and/or improve cardiac work in rats and mice with experimental left descending coronary (LAD) occlusion followed by reperfusion [12,13,14]. Similarly, in the acute phase of ischemia/reperfusion, the single intravenous (i.v.) injection of CBD mainly before ischemia and sometimes also before reperfusion in rats [15,16] and in rabbits [17] not only reduced the infarct size and increased the blood flow [15,17] but also acted antiarrhythmic [15,16] and inhibited platelet aggregation [15]. Only Durst et al. [12] failed to notice alterations in infarct size and cardiac function ex vivo in hearts isolated 24 h and 1 h after CBD administration and subjected to ischemia/reperfusion. However, one should keep in mind that cardiac ischemia/reperfusion damage can also affect other organs, including the brain. It is noteworthy that CBD acted protectively against brain injury [18] and lung injury [19] resulting from cardiac ischemia/reperfusion.

The results of preclinical studies suggested that the well-known properties of CBD, including the regulation of inflammation, oxidative stress, and fibrosis, along with following other potential mechanisms, may be responsible for the beneficial effects of CBD in ischemia/reperfusion injury [13,14,15,16] and improve the function of the coronary arteries or the myocardium. CBD is known for its vasodilatory activity observed in human mesenteric [20] or pulmonary [21] arteries, among others. Nevertheless, we demonstrated that chronic CBD administration failed to modify coronary perfusion pressure at rest. However, it reduced the carbachol-induced vasoconstriction of coronary arteries, increased the width of ventricular cardiomyocytes, and exerted beneficial effects on cardiac and plasma oxidative stress in spontaneously hypertensive rats (SHR) rats [22,23]. The beneficial anti-inflammatory influence of CBD has been determined in human coronary artery endothelial (HCAECs) and smooth muscle (HCASMC) cells [24,25].

Additionally, CBD might also modify the function of cardiac muscle. Thus, its acute administration decreased the duration of the action potential in rat ventricular myocytes [26] or inhibited multiple ion channels in rabbit ventricular cardiomyocytes [27]. Moreover, increasing concentrations of CBD decreased the contractility in isolated rat atria from both SHR and their normotensive Wistar Kyoto control rats (WKY) [28]. Furthermore, CBD enhanced the increase in contractility of rat left atria induced by the nonselective β-adrenoceptor agonist isoprenaline (ISO) but failed to modify the positive chronotropic effect of isoprenaline in rat right atria [29,30]. On the other hand, chronic treatment with CBD did not affect the inotropic response to isoprenaline in isolated left atria and its chronotropic and inotropic response in isolated hearts of SHR and their normotensive control [22].

Pharmacological modulation of β-adrenoceptors is suggested as a new cardioprotective strategy for therapy of myocardial dysfunction induced by ischemia and reperfusion [31,32,33]. Indeed, activation of β-adrenoceptors mimics preconditioning of isolated rat atria and ventricles against ischemic contractile dysfunction [34], in rat perfused hearts [35], and in papillary muscles isolated from normal and postinfarction rat hearts [36]. Moreover, isoprenaline given prior to ischemia attenuated the subsequent postischemic ventricular dysfunction in hearts isolated from younger but not old SHR rats [37].

However, there are significant atrial and ventricular differences regarding their mechanical and electrical functions, or their biochemistry and histology [34,38,39,40,41,42].

In light of the aforementioned considerations, the objective of our study was to: (1) a comparison of hypoxia/reoxygenation (H/R) influence on the function of right and left atria (RA and LA) as well as in papillary muscle from right and left ventricle (RV and LV) under basal conditions and after previous stimulation with isoprenaline; (2) the examination of the influence of acute CBD administration on hypoxia/reoxygenation induced changes in work and on cardiostimulatory effect of isoprenaline in all 4 cardiac compartments; (3) assessment of chronic CBD administration on hypoxia/reoxygenation-induced changes and on positive inotropic isoprenaline effect in left atria isolated from SHR and WKY rats.

## 2. Results

### 2.1. General

**Figure 1:**
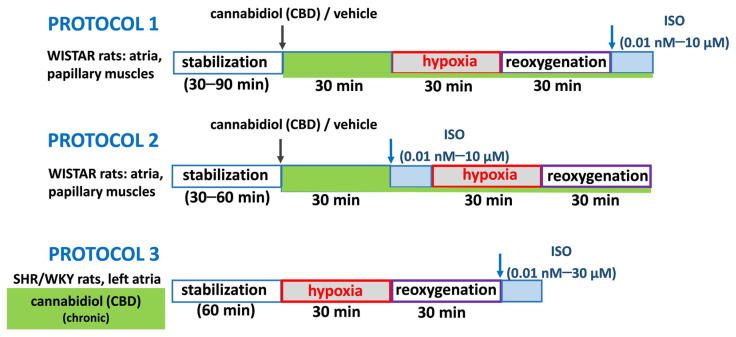
Experimental protocols used in the current studies. In acute experiments on cardiac tissues collected from Wistar rats, after 30-min incubation with 1 µM cannabidiol (CBD) or its vehicle, in accordance with Protocol 1, tissues were exposed to 30 min hypoxia and 30 min reoxygenation with followed increasing concentrations of the nonselective β-adrenoceptor agonist isoprenaline (ISO) administration (0.01 nM–10 µM). In Protocol 2, after incubation with CBD/vehicle tissues were exposed to ISO (0.01 nM–10 µM) and then to H/R. In chronic experiments, left atria isolated from spontaneously hypertensive rats (SHR) and their normotensive control (WKY) rat 24 h after 2-week treatment with CBD 10 mg/kg/day or its vehicle were subjected to H/R with following ISO administration (0.01 nM–30 µM, Protocol 3).

As detailed in Material and Methods, as illustrated in Figure 1, three experimental protocols were applied. Shortly, in acute experiments after administration of CBD (1 µM) or its vehicle, the concentration curves for isoprenaline were constructed after (protocol 1) or before (protocol 2) hypoxia (30 min) and reoxygenation (30 min). In chronic experiments (protocol 3), left atria isolated from SHR and their normotensive control (WKY) rats collected 24 h after 2-week treatment with CBD 10 mg/kg/ days or its vehicle were subjected to hypoxia/reoxygenation with following isoprenaline administration.

As shown in Table 1, in experiments with acute administration of CBD (both according to Protocols 1 and 2), all basal values (rate of spontaneously beating right atria and force of stimulated left atria and papillary muscles isolated from Wistar rats) immediately before and 30 min after administration of CBD (1 µM) were comparable (Table 1).

### 2.2. Influence of β-Adrenergic Stimulation and Cannabidiol on Time-Dependent Hypoxia-Reoxygenation-Induced Changes in Rate and Force of Contractions

**Figure 2:**
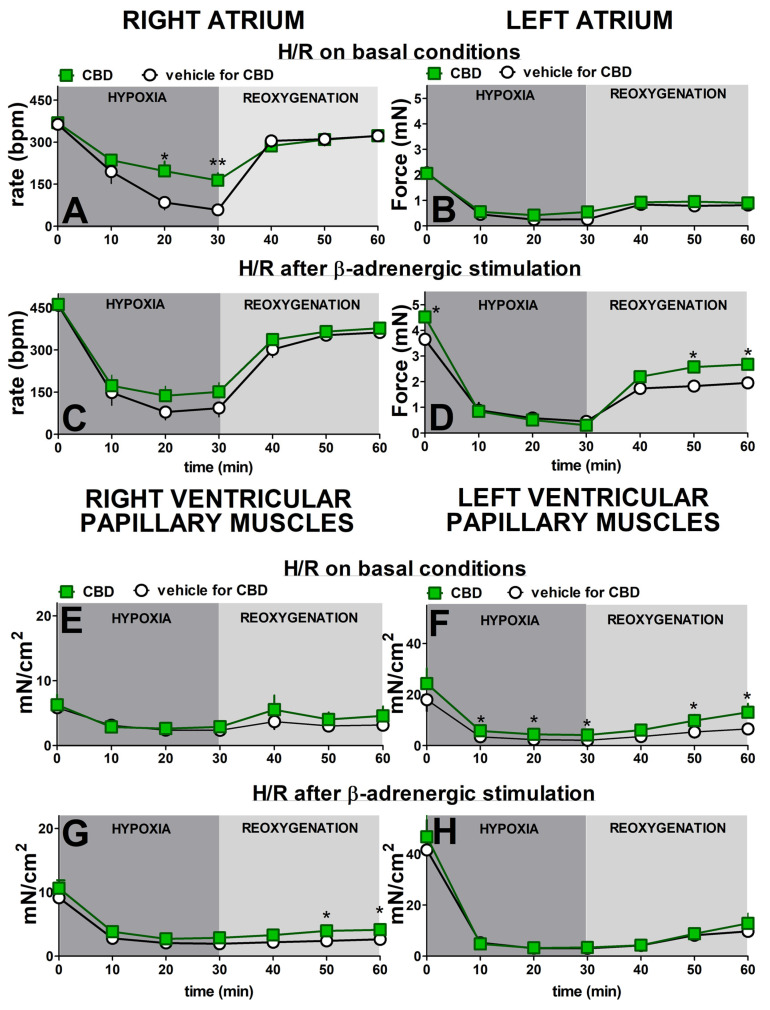
Influence of β-adrenergic stimulation and cannabidiol (CBD) on time-course of hypoxia and reoxygenation (H/R) in cardiac preparations isolated from Wistar rats. Experiments were performed in basal conditions (**A**,**B**,**E**,**F**) or under previous stimulation with β-adrenergic agonist isoprenaline (0.1 nM–10 μM) (**C**,**D**,**G**,**H**). Cannabidiol (CBD) 1 µM or its vehicle was administered 30 min before the beginning of hypoxia (**A**,**B**,**E**,**F**) or before the first concentration of ISO (**C**,**D**,**G**,**H**). For details of protocols, see Figure 1. Data are given as the means ± SEM of 6–10 rats. *t*-test * *p* < 0.05, ** *p* < 0.01; significant effect of CBD. Student′s *t*-test for nonpaired data; bpm, beats per minute.

After 30 min of hypoxia, all cardiac preparations showed a decrease in contraction rate and force (Figure 2). In the absence of previous β-adrenergic stimulation, the greatest decrease in rate of contraction of the right atria (to approximately 15% of initial values) took place 30 min after the beginning of hypoxia. Reoxygenation gradually increased this parameter to nearly 90% of the prehypoxic levels after 30 min (Figure 2A). Isoprenaline increased the prehypoxic rate of contraction approximately by 30% (Figure 2C). Despite higher basal values, it only slightly slowed (in comparison to Figure 2A) the decline in rate during hypoxia in the right atrium to approximately 20% of the initial values. Reoxygenation, however, restored the rate to that observed in atria that had not been prestimulated with isoprenaline (Figure 2C).

Hypoxia significantly reduced the contraction force in left atria (Figure 2B,D), right (Figure 2E,G), and left (Figure 2F,H) ventricular papillary muscles in a similar manner both in the absence (Figure 2B,E,F) and in the presence (Figure 2D,G,H) of previous β-adrenergic stimulation. Thus, under control conditions, hypoxia diminished the contraction force to 10, 40, and 10% of initial values, respectively. In almost all cases, the maximal changes were evident after the first 10 min of hypoxia and persisted until reoxygenation began. The force in the left atria and right and left ventricular papillary muscles returned to approximately 45, 55, and 35% of their prehypoxic levels at the end of recovery.

Prestimulating the left atria and right and left ventricular papillary muscles with isoprenaline increased initial values by approximately 80, 55, and 130%, respectively, compared to the respective nonstimulated groups. Previous β-adrenergic stimulation did not modify the time-course and degree of the hypoxia-induced changes in none of the above cardiac compartments (Figure 2C,G,H). Thus, all maximal decline in contraction by approximately 75–90% of prehypoxia values took place in the first 10–20 min after hypoxia. The increase in basal values by isoprenaline slightly improved the reoxygenation-elicited recovery in the left atrium (Figure 2C) to approximately 55% of its initial values in comparison to the 40% noticed under basal conditions (Figure 2A). We were unable to compare these changes statistically because of significant differences in basal values. In contrast to the left atrium, the previous β-adrenergic stimulation did not substantially modify the time course and degree of the reoxygenation-induced changes in right and left ventricular papillary muscles (Figure 2G,H).

CBD 1 µM significantly prevented the hypoxia-induced decrease in contraction rate in right atria, but only in the absence of previous β-adrenergic stimulation (Figure 2A,C). Thus, the maximal fall in rate was approximately 85 and 55% of prehypoxic values in the control and treated with CBD groups, respectively (Figure 2A). CBD did not modify the recovery rate whether it was examined with or without β-adrenergic prestimulation (Figure 2A,C).

CBD 1 µM also improved contraction recovery (by approximately 10%) in the left atrium (Figure 2D) and in the right ventricular papillary muscles (Figure 2G), but only in the presence of previous β-adrenergic stimulation. On the contrary, the comparable degree of contraction recovery improvement in left ventricular papillary muscles took place only under basal conditions, i.e., in the absence of previous isoprenaline application (Figure 2F). The small (by approximately 20%) but significant beneficial effect of CBD 1 µM on the hypoxia-induced fall in contractility was visible in left ventricular papillary in the absence (Figure 2F) but not in the presence (Figure 2H) of previous β-adrenergic stimulation. It was also not observed independent of experimental conditions in the left atrium (Figure 2B) and in the right ventricular papillary muscles (Figure 2E).

### 2.3. Influence of Acute Treatment with Cannabidiol and Hypoxia/Reoxygenation on Isoprenaline-Induced Cardiostimulatory Effects

**Figure 3:**
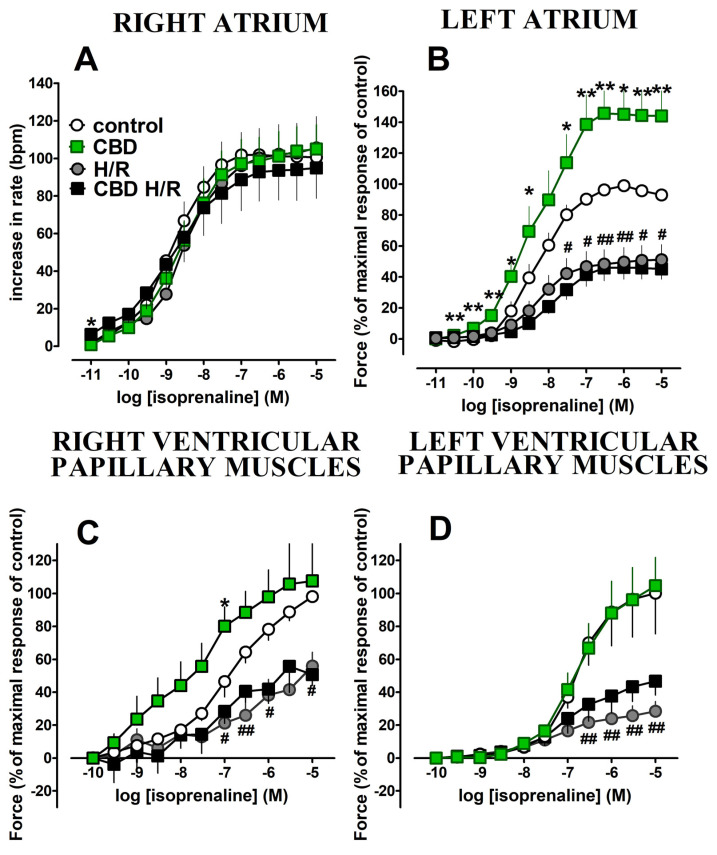
Influence of cannabidiol (CBD; 1 µM) on the isoprenaline (ISO)-induced changes in rate ((**A**), right atria) and force of contractions ((**B**), left atria and (**C**,**D**), ventricular papillary muscles) isolated from Wistar rats. The concentration curves for isoprenaline were constructed before (control, CBD) or after hypoxia and reoxygenation (H/R, CBD H/R). Cannabidiol (CBD) 1 µM or its vehicle was administered 30 min before the beginning of hypoxia (H/R, CBD H/R) or before the first concentration of ISO (control, CBD). For experimental protocol, see Figure 1. Values were expressed as an increase in the rate of the right atria and as percentages of the maximum responses to isoprenaline (left atria and papillary muscles) of the control group. Data are given as the means ± SEM of 6–10 rats. * *p* < 0.05, ** *p* < 0.01; significant effect of CBD. ^#^ *p* < 0.05, ^##^ *p* < 0.01 significant effect of H/R. One-way analysis of variance (ANOVA) with Bonferroni post hoc test. bpm, beats per minute.

Isoprenaline (0.1 nM–10 μM) caused concentration-dependent increases in the rate of contractions in the right atrium (Figure 3A) and force of the left atrium, and right and left ventricular papillary muscles (Figure 3B–D). Hypoxia/reoxygenation did not influence the positive chronotropic effect of isoprenaline in the right atria (Figure 3A) but strongly diminished the positive inotropic effects of isoprenaline in the left atria and right and left ventricular papillary muscles (decreases in E_max_ were approximately by 50, 40, and 70% in comparison to the control, respectively) (Figure 3B–D). However, the potencies of isoprenaline were not affected (Table 1).

Cannabidiol 1 μM strongly enhanced the positive inotropic effect of isoprenaline in the left atrium; E_max_ was increased by approximately 45% in comparison to the control group (Figure 3B, Table 1). In the right ventricular papillary muscles, CBD tended to increase the response to isoprenaline and significantly enhanced its positive inotropic effect at higher (0.1 μM) concentrations (Figure 3C). However, the potency of isoprenaline and E_max_ were not affected (Table 1). The positive chronotropic and inotropic effects of isoprenaline in the right atria and left ventricular papillary muscles, respectively, were unmodified by CBD (Figure 3A,D).

Hypoxia/reoxygenation abolished the enhancing effect of CBD on the positive inotropic effect of isoprenaline in the left atrium (Figure 3B; Table 1,) and in the right papillary muscles (Figure 3C; Table 1). Cannabidiol did not modify the diminished by hypoxia/reoxygenation chronotropic and inotropic action of isoprenaline in the right atrium and in the left ventricular papillary muscles, respectively (Figure 3A,D and Table 1).

### 2.4. Influence of Chronic Treatment with CBD on the Hypoxia/Reoxygenation-Induced Changes and Cardiostimulatory Effect of Isoprenaline in Left Atria Isolated from HR and WKY Rats

**Figure 4:**
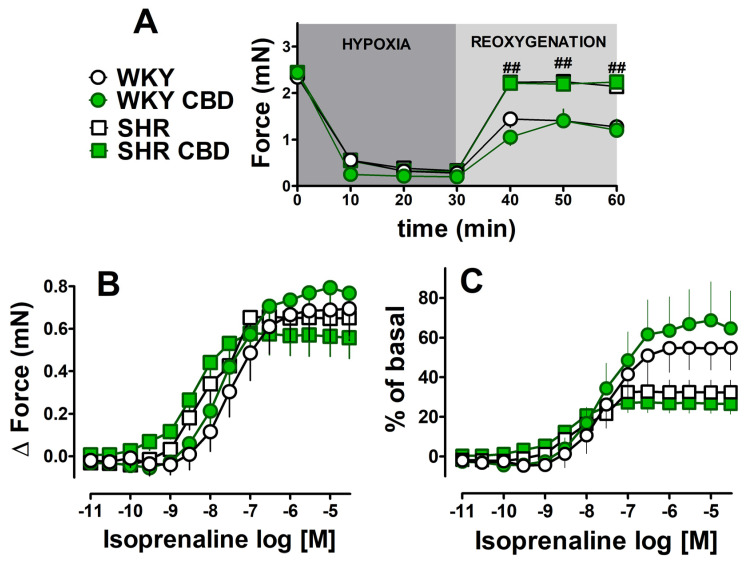
Influence of chronic treatment with cannabidiol (CBD) (every 24 h for 14 days, i.p., 10 mg/kg) on the force of contractions of atria isolated from spontaneously hypertensive (SHR) rats and their normotensive control (WKY) undergoing hypoxia/reoxygenation (H/R) with followed stimulation with isoprenaline (ISO; 0.01 nM–30 μM). (**A**) time course of hypoxia and reoxygenation; positive inotropic response to ISO: (**B**) expressed as changes in force (delta) of contractions, (**C**) expressed as percentages of basal values. Data are given as the means ± SEM of 5–7 rats. ^##^ *p* < 0.001; SHR significantly different from WKY, one-way analysis of variance (ANOVA) with Bonferroni post hoc test.

Basal force of contractions of left atria isolated from spontaneously hypertensive rats and their normotensive controls (WKY) (Table 2) were comparable to respective values in Wistar rats (Table 1). Chronic administration of CBD (every 24 h for 14 days, i.p., 10 mg/kg) failed to affect basal values both in SHR and in WKY (Table 2). Hypoxia decreased the force of contractions of the left atria in a comparable manner both in SHR and WKY (Table 2, Figure 4A) and in Wistar rats (Figure 2) (to approximately 90% of prehypoxic parameters). Interestingly, SHR atria demonstrated a significant, rapid improvement in recovery (noticeable within the first 10 min and observed until the end of the recovery period) when compared to WKY rats (by approximately 85% and 50% of prehypoxic values, respectively), i.e., to 2.1 ± 0.16 (*n* = 7) and 1.3 ± 0.16 (*n* = 5) mN (*p* < 0.05). As a result, the basal values immediately before administration of the first concentration of isoprenaline were almost two times higher in atria isolated from SHR than from WKY (Figure 4A). The chronic treatment of SHR and WKY rats with CBD failed to prevent the decline or improve recovery in the atria (Figure 4A).

After hypoxia/reoxygenation, the β-adrenergic stimulation with increasing concentrations of isoprenaline (0.01 nM–30 μM) was started (for details see Figure 1). Since basal values of atrial contractions (after hypoxia/reoxygenation) differ between WKY and SHR rats, data were expressed both, as changes (delta) in the force of contractions and as percentages of basal values (Figure 4B and 4C, respectively). When data are expressed as changes in contraction force (delta), maximal responses to isoprenaline were similar despite higher basal values noticed in atria isolated from SHR than from WKY (see the previous paragraph), but hypertension slightly enhanced the potency of isoprenaline (Table 2). As a result, the inotropic effect of isoprenaline (expressed in % of basal values) tended to be higher in WKY, where basal contractility was lower than in SHR. Hypertension tended to decrease the maximal response to isoprenaline (E_max_ reduced approximately 40% when expressed as % of basal) (Figure 4C; Table 2). For comparison, the maximal isoprenaline-induced increase in the force of contractions of the left atria isolated from Wistar rats before hypoxia/reoxygenation was approximately 1.5 mN (expressed in % of controls in Figure 3). Chronic CBD administration did not modify the inotropic effect of isoprenaline in left atria isolated from WKY and SHR rats (Figure 4B,C).

## 3. Discussion

### 3.1. General

The aim of the present study was to compare the impact of hypoxia/reoxygenation in the absence and presence of β-adrenergic stimulation by the nonselective β-adrenoreceptor agonist isoprenaline on cardiac tissues representing all four heart compartments: the right and left atria and the papillary muscles of both the right and left ventricles. In addition, our objective was to determine how CBD affects the heart muscle directly in response to alterations induced by the cardiac effects of hypoxia/reoxygenation and isoprenaline. Thus, we used a coronary-vascular independent model to examine CBD′s direct effects separately on the rate and force of contractions in different heart regions. Examination of the right atria allowed us to assess changes in spontaneous beating rate. The modification of contractility was examined in experiments on the electrically stimulated left atria and papillary muscles of both ventricles, where the constant contraction frequency did not interfere with the registered parameter. The cardiac papillary muscle has been a fundamental experimental model for the study of ventricular muscle physiology for decades [43]. Unlike a muscle strip isolated from the ventricular wall, the papillary muscle may be prepared in its entirety without significant damage [44]. In acute experiments, we have used CBD at a concentration of 1 µM, as this concentration has been demonstrated to modulate contractility and excitation-contraction coupling in cardiomyocytes [26], promote the proliferation of neonatal cardiomyocytes in vitro [14], and, based on our previous findings, enhance the positive inotropic effect of isoprenaline in isolated left atria [29]. Using CBD at 10 mg/kg for chronic experiments has previously demonstrated beneficial effects on cardiovascular tissues, including cardiac parameters in SHR (for details, see Introduction, [22]). Additionally, it has been shown to reduce oxidative stress in diabetic rats and mice [45,46], septic rats [47], doxorubicin-induced cardiomyopathy in mice [48], and experimental autoimmune myocarditis [49].

### 3.2. Influence of Hypoxia/Reoxygenation on the Function of Four Cardiac Compartments and the Significance of β-Adrenergic Stimulation

In our experiments, hypoxia caused an immediate and significant (by up to 90%) decline in both the rate and force of contraction in all examined cardiac tissues. However, our experiments revealed that the inhibition of rate in the right atria occurred more slowly than in the case of contractility (in the right atria and in the right and left ventricular papillary muscles), since the maximal effect of hypoxia was observed within 20 and 30 min after its introduction, respectively. Similarly, we noticed a different time course of reoxygenation. Thus, the right atrial rate returned almost to basal values within 10–20 min following the initiation of reoxygenation. On the contrary, contractility (in the left atria and in both ventricular papillary muscles) was still strongly diminished (by approximately 25–70%) at the end of the experiments (30 min reoxygenation). We suggest that the different patterns of rate and contractility responses to hypoxia/reoxygenation do not originate from disparate cardiac compartments but rather from the potential for the right atria to slow rate (and thus simultaneously reduce oxygen demand) in response to hypoxia. Such possibility was excluded in electrically stimulated left atria and in both ventricular papillary muscles. To the best of our knowledge, this study is the first one, in which the effects of hypoxia and reoxygenation were examined in four chambers of the heart, including the influence on right atrial beating rate. Only limited data are available from experiments conducted at lower, nonphysiological temperatures using different buffer compositions (including 20% oxygen). Although the authors observed a decrease in contractility, they stated that there was no change in the beating rate (without providing data) [50]. Similarly, hypoxia shortened the duration of the action potential in left but not in right rabbit atria [51].

As outlined in the Introduction, pharmacological modulation of β-adrenoceptors is suggested as a new cardioprotective approach for the treatment of myocardial dysfunction resulting from ischemia and reperfusion [31,32,33]. Accordingly, we have applied experimental protocols that assumed the evaluation of the cardiac preparation’s response to hypoxia/reoxygenation during resting baseline and preconditioned with the nonselective β-adrenoceptor agonist isoprenaline. Isoprenaline increased basal rate and contractility in all four examined cardiac tissues, leading to enhancement of the basal parameters of contractility before hypoxia. In spite of the above-mentioned differences in the basal parameters before hypoxia, in our hands, previous β-adrenergic stimulation improved only the cardiac recovery in the left atrium but not in the papillary muscles from the right and left ventricles. Thus, we confirmed the previous observations that preconditioning with isoprenaline only in rat-isolated left atria showed improved recovery of developed tension 60 min after reoxygenation, but not in right ventricular strips [34]. The similar effects were obtained instead of the fact that Penson et al. [34] used a lower and single concentration of isoprenaline (0.1 µM), and we used rising concentrations of isoprenaline to evaluate the ability of damaged cardiac tissues to counteract the conditions that have forced them to work harder. Interestingly, the beneficial effect of isoprenaline was noticed in left atria stimulated electrically with a steady rate but not in right atria, in which the rate of contraction could be adapted to a lower oxygen supply. In the right atria, however, full recovery was achieved independently of isoprenaline use.

The beneficial effects of β-adrenergic stimulation have been mainly demonstrated previously in experiments on isolated hearts. Thus, isoprenaline-provided preconditioning was able to mimic ischaemic preconditioning and resulted in a better recovery of left ventricular developed pressure, a decrease in creatine kinase release, and an improvement in the coronary flow of ischemic isolated rat hearts [35,37,52,53]. The mentioned above beneficial influence of isoprenaline was suppressed by nonselective β-adrenoceptor antagonists (propranolol and atenolol) [34,47,52,53] confirming the involvement of β-adrenoceptors in these effects. However, the β_2_-adrenoceptor antagonist ICI 118551 failed to modify the favorable effects of isoprenaline on creatine kinase release and coronary flow [52], suggesting complex mechanisms underlying isoprenaline cardioprotection against hypoxia/reoxygenation. One should also keep in mind that the β_1_-adrenoceptor stimulation increases the oxygen consumption in the heart and might exert detrimental effects during ischaemia and reperfusion, leading to further enhancement of myocardial injury due to its positive inotropic and chronotropic activities, whereas β_2_-adrenoceptors might have protective postconditioning actions (according to [32]). Thus, the lack of beneficial effects of isoprenaline in our study in right atria, and in both ventricular papillary muscles might result from (1) the interplay between opposite effects of β_1_- and β_2_-adrenoceptors in particular regions of the heart or (2) from the dependence of such effects on myocardial coronary flow, but not from the direct action of isoprenaline on cardiac tissue [33].

### 3.3. Potential Beneficial Influence of CBD against Hypoxia/Reoxygenation Injury in Four Cardiac Compartments

We demonstrated that cannabidiol exerted beneficial effects against hypoxia/reoxygenation in the manner dependent on heart region and previous β-adrenergic stimulation. Thus, it diminished the consequence of the hypoxia-induced fall in right atrial rate but only in the absence of previous isoprenaline application. Moreover, it has been shown to improve recovery in the left atria and in right ventricular papillary muscle but only in the presence of previous β-adrenergic stimulation. On the other hand, in the left ventricular papillary muscles, the beneficial influence of CBD on the recovery of contractility was evident only without prior β-adrenergic stimulation. It has been demonstrated previously, that different effects on particular cardiac chambers might result from: differential phosphodiesterase enzymes (PDE1–5) mRNA and protein expressions [38], differences in the fundamental mechanisms of contractility regulation and calcium handling [39], the duration of isometric contraction, Ca^2+^ transients and action potential [40], post-translational modifications affecting channels function [41], difference in the ratio of α-myosin and β-myosin isoforms [42], and finally relative proportions of the two β-adrenoceptor subtypes in cardiac tissue [54]. We assume that pressure measurements within a specific region of the heart might be responsible for mentioned above cardiac effects of CBD effects. Left atrium and right ventricle are low-pressure chambers whereas the left ventricle is a high-pressure chamber. It might be too low in low-pressure chambers (left atria and right ventricle) and should therefore be enhanced by β-adrenergic stimulation first. On the other hand, it might be too high in high pressure chambers (left ventricle) after additional stimulation by isoprenaline. In this context, CBD has a protective effect in the right atrium at low beating rate at baseline, but this effect was diminished when the beating rate was increased by isoprenaline.

In our experimental model, we noticed a direct influence of CBD on the contractility of cardiac muscle preparations. A majority of previous studies examined the potential cardioprotective properties of the CBD against ischemia/reperfusion in isolated hearts and in experiments in vivo. As aforementioned in the Introduction, CBD, given in in vivo, significantly reduced the infarct size and improved the cardiac performance [12,13,15,17] via its anti-inflammatory [12,17] or antiarrhythmic effects [15,16] as well as via inhibition of platelet aggregation [15]. In addition, CBD decreased the necrotic zone and increased blood flow in the area at risk [12,17]. It has been suggested that other CBD′s well-known properties such as regulating inflammation, oxidative stress, and fibrosis may be responsible for its beneficial effects on ischemia/reperfusion injury [13,14,15,16]. Concerning the protective effect of CBD against hypoxia-induced falls in rate of contractions, one might assume that CBD affects cardiac electrophysiology by acting on a diverse range of ion channels. However, the reported effects of CBD on cardiac channels are contrary and depend on cardiac tissue origin. Thus, CBD (1 µM) was shown to shorten action potential duration in rat ventricular myocytes [26] and in rabbit Purkinje fibers [55] and increased it in rabbit and guinea pig right ventricular papillary muscles [56]. At higher concentrations (5 µM), CBD increased the action potential duration in rabbit and dog ventricular papillary muscles [57] by affecting major cardiac ion currents such as Na^+^ [55,56], L-type Ca^2+^ [26,27,55] and K^+^ [55,56,57]. Here, we could exclude CBD-dependent changes in the action potential as the underlying mechanism by which CBD influences the contractility, since left atria and both papillary muscles were paced with constant parameters [58].

### 3.4. Influence of Hypoxia/Reoxygenation and CBD on Cardiostimulatory Effects of Isoprenaline in Four Cardiac Compartments

We confirmed the previous observations [29,30] that CBD enhanced the positive inotropic effect of isoprenaline in the left atrium and failed to modify the positive chronotropic action of isoprenaline in the right atrium. In addition, it increased the force of contraction in the left papillary muscles and improved contractility response to isoprenaline in the right ventricular papillary muscles, although the effect in the right ventricle was not as evident as in the left atria. Importantly, also in the case of the influence of CBD, we noticed the differences between low- and high-pressure compartments since CBD did not modify the inotropic action of isoprenaline in papillary muscles from the left ventricle at all. How can we explain the modulatory effect of CBD on the isoprenaline-induced increase in the force of contraction? We suggest the following possibilities. Firstly, the potential involvement of cannabinoid CB_1_ receptors in the amplificatory influence of CBD on the positive inotropic action of isoprenaline, since CBD is known as a negative allosteric modulator of CB_1_ receptors [59] and stimulation of CB_1_ receptors decreased contractility of isolated rat left atria [60]. A complex interaction was demonstrated between CB_1_ receptors and β_2_-adrenoceptors-pERK signaling in human embryonic kidney and primary human trabecular meshwork cells [61]. Secondly, acute CBD administration possessed an antiarrhythmic effect against ischemia reperfusion-induced arrhythmias in anesthetized rats via the activation of adenosine A1 receptors [16]. Interestingly, adenosine A1 receptors have been demonstrated to heterodimerize with β_1_- and/or β_2_-adrenocptors, which leads to altered receptor pharmacology, functional coupling, and intracellular signaling pathways [62]. On the other hand, another plant-derived cannabinoid, namely delta 9-tetrahydrocannabinol (but not cannabidiol), increased β-adrenergic receptor binding in mouse cerebral cortex [63].

We demonstrated previously that CBD at concentrations ranging from 1 nM to 30 μM decreased contractility in the left atria isolated from WKY and SHR, maximally by approximately 20% [28]. However, in the current study, 1 μM CBD failed to modify the basal contractility in all cardiac regions and the rate of right atria, which is consistent with our previous results obtained on cardiac tissue isolated from Wistar rats [29]. This difference might result from the different rat strains (WKY vs. Wistar) or the route of CBD administration (the whole concentration-response curve vs. one dose). Importantly, we can exclude the possibility that various basal values are responsible for different influences of CBD on the region-specific cardiostimulatory action of isoprenaline.

Hypoxia/reoxygenation strongly diminished the positive inotropic effects of isoprenaline both in the left atria and in the right and left ventricular papillary muscle but did not modify the chronotropic action of isoprenaline in the right atria. Our results are consistent with those of previous studies conducted in isolated electrically stimulated mouse left atria subjected to hypoxia [64] but also demonstrate that similar effects are observed in other compartments, but not in the right atrium. CBD did not affect the cardiostimulatory action of isoprenaline changed by hypoxia/reoxygenation in any cardiac region. We can only suppose that it might result from too low basal values.

### 3.5. Influence of Chronic Administration of CBD on Changes Induced by Hypoxia/Reoxygenation and Isoprenaline in SHR and WKY Rats

We demonstrated that left atria isolated from spontaneously hypertensive rats exhibited comparable sensitivity to hypoxia but demonstrated superior force recovery after reoxygenation in comparison to their normotensive controls (WKY). To the best of our knowledge, no previous studies have directly compared the effects of hypoxia/reoxygenation directly on cardiac tissue isolated from hypertensive animals. The previously reported findings regarding acute myocardial ischemia/reperfusion are derived mostly from in vivo studies or experiments on isolated hearts with a fully functioning coronary system or wider systemic control. Their final conclusions are different and mainly dependent on the experimental model. In contrast to our observation, in comparison to the respective normotensive controls, the hearts of SHR were more vulnerable to ischemic injury [65]. On the other hand, no significant increase in infarct sizes was noticed in hypertrophied hearts of Dahl salt-sensitive hypertensive rats [66] or SHR [67], while other studies have shown that diabetic animals [68], those with angiotensin II-dependent hypertension [69], or those with two-kidney one-clip Goldblatt hypertension [70] exhibit even smaller cardiac infarct sizes in response to acute myocardial ischemia/reperfusion in comparison to respective normotensive controls. In our study, we showed improvement in the force of isolated left atria during reoxygenation, but we were not able to determine the infarct size in our model.

In the left atria that underwent previous hypoxia/reoxygenation, we obtained identical concentration-response curves for inotropic effects (expressed in mN) of isoprenaline in tissues isolated from SHR and WKY. It should be noted, however, that the basal force tone immediately before an administration of the first concentration of isoprenaline was almost two times higher in SHR than in WKY, causing the inotropic effects of isoprenaline (expressed in % of basal values) to tend to be higher in WKY than in SHR. This final observation is in accordance with our previous studies, in which we demonstrated that the positive inotropic effects of isoprenaline in left atria (under control conditions with comparable basal force) isolated from SHR were reduced in comparison to the respective responses in WKY [22].

In our current study, we demonstrated that in contrast to the acute application of CBD, its chronic administration (every 24 h for 14 days, i.p., 10 mg/kg) failed to improve both, resistance to hypoxia and recovery after hypoxia in left atria isolated from hypertensive SHR rats and their normotensive WKY control. Importantly, we noticed additionally that the pronounced amplifying effect of acute CBD administration on the inotropic action of isoprenaline in left rat atria was absent after chronic administration.

We were able to examine only left atria since results were obtained on isolated hearts or the fragments we described in our previous publications, in which we also justified the use of chronic CBD dose and experimental model of hypertension [22,23,71]. However, the left atrium plays a crucial role in determining the left ventricle′s filling and, therefore, cardiac output. An impaired left atrial function may result in impaired left ventricle performance, resulting in heart failure [72]. Thus, the current work extended our previous observations, in which we demonstrated that chronic CBD treatment reduced the carbachol-induced vasoconstriction of coronary arteries, increased the width of ventricular cardiomyocytes, and exerted beneficial effects on cardiac and plasma oxidative stress in SHR rats [22,23]. Furthermore, chronic administration of CBD prior to ischemia was observed to reduce infarct size in both anesthetized rats [12] and mice [14].

**Figure 5:**
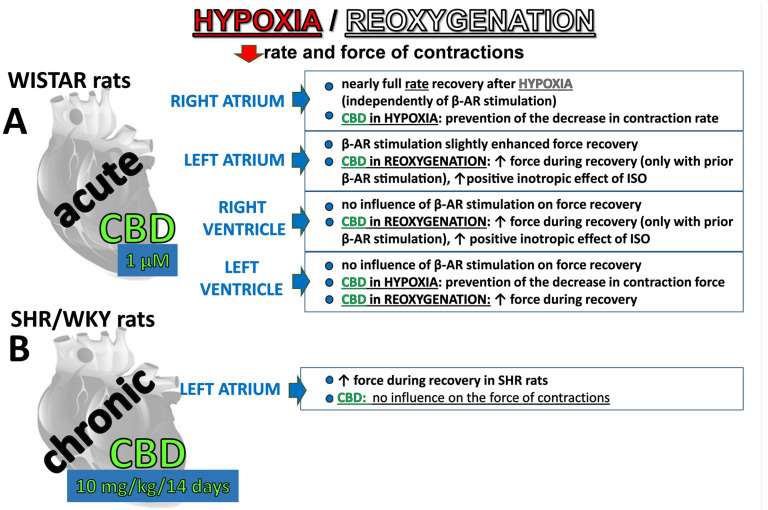
Summary of major effects of hypoxia/reoxygenation as well as β-adrenergic (β-AR) stimulation and cannabidiol′s (CBD)—mediated cardioprotection in rat cardiac tissues isolated from four cardiac compartments (for details of protocols, see Figure 1). CBD was administered acutely (**A**) to organic baths containing cardiac samples or chronically (**B**) to spontaneously hypertensive rats (SHR) and their normotensive controls (WKY) and isolated left atria were subjected to hypoxia/reoxygenation.

Based on the results of our experiments with acute and chronic CBD administration (Figure 5), it can be postulated that the beneficial effects of CBD on hypertension are more closely associated with its ability to reduce vascular dysfunction, inflammation, and oxidative stress than with an improvement in cardiac muscle contractility [12,15,16,23].

The question arises what is the clinical relevance of our study in light of the lack of the beneficial cardioprotective influence of chronic CBD use against cardiac hypoxia in hypertension? From one side, it should be noted that other results may be obtained under in vivo conditions or in experiments performed on isolated whole hearts (described in detail in the Introduction), since our results indicate the majority role of coronary flow and systemic factors in CBD cardioprotective effects. The age of the SHR could also have an impact on our results, as we used rats at the early stage of hypertension (8–9 weeks) that did not develop remodeling of the left atrium and subsequent heart failure [72]. The vasodilatory potency of CBD was dependent on the model of hypertension; it was more potent in deoxycorticosterone plus salt treatment (DOCA-salt) hypertension, but was reduced in SHR [21], and we cannot exclude the beneficial effects of CBD in other hypertensive models. On the other hand, we did not notice any cardiac adverse effects of CBD on H/R. In contrast, chronic CBD administration increased cardiac lipid peroxidation and decreased β_1_-adrenoceptor densities in normotensive controls [22,23].

## 4. Strengths and Limitations

We examined the effects of 30 min hypoxia followed by 30 min reoxygenation in the absence and presence of increasing concentrations of isoprenaline and/or CBD (1 µM) directly on cardiac tissues, i.e., on rate of contraction in right atria and the force of contraction in left atria and papillary muscles of both right and left ventricles. Our study had the main following strengths: (1) the study was conducted on four compartments of the heart independently to assay their potential different reactions based on their various functions; (2) the direct effects of CBD on the myocardium were examined under conditions of hypoxia and reoxygenation, extending the scope of previous experiments conducted on vascular-dependent models only; (3) investigating CBD′s effect in conjunction with β-adrenergic receptor stimulation since such stimulation has been shown to improve cardiac tolerance to ischemia; (4) comparison of the cardioprotective potential of CBD given in an acute and chronic manner; (5) examination of the hypertensive myocardium response in comparison to normotensive tissues against hypoxia and reoxygenation.

However, our study has also some limitations. It would be interesting (1) to also examine changes in the contractility of the right atrium, since hypoxia diminished this parameter in the right atrium without changing the sinus rate [50]; unfortunately, the acquisition system we used allowed us to determine only the rate or force, and adding more animals would not be consistent with the principles of the 3Rs, (2) to apply other study protocols, primarily with a longer period for reoxygenation and administration of CBD after hypoxia, i.e., in the therapeutic manner; (3) and one should note that in our experimental setup, we were unable to measure infarct size. Moreover, using isolated cardiac tissue preparations eliminates any factors that affect myocardial infarction in vivo, including vascular function, neurohumoral stimulation, and physical and metabolic stress. (4) This study was designed to examine the functional effects of CBD in cardiac preparations in vitro. As a result, it is descriptive in nature and lacks experiments that would examine the underlying mechanisms of the CBD effects. Unfortunately, this was beyond the scope of the present work and has to be addressed in future research.

## 5. Materials and Methods

Figure 1 illustrates three different protocols used for our experiments. The first two protocols concern acute experiments on cardiac tissues collected from Wistar rats. According to protocol 1, 1 µM CBD/DMSO was added to the organ baths 30 min before the initiation of hypoxia and reoxygenation with followed ISO administration (0.01 nM–10 µM). In protocol 2, after 30-min incubation with 1 µM CBD (or its vehicle DMSO), tissues were exposed to increasing concentrations of the nonselective β-adrenoceptor agonist isoprenaline (ISO) (0.01 nM–10 µM) similarly to our previous study [22] and then to 30 min hypoxia followed by 30 min reoxygenation. CBD was left in organ baths during H/R or/and isoprenaline addition. For experiments where CBD was given chronically (SHR/WKY rats), protocol 3 was used, and left atria were conducted to H/R with the following ISO administration (0.01 nM–30 µM).

### 5.1. Animals

Experiments were performed on male Wistar rats (290–360 g), spontaneously hypertensive rats (SHR) weighing 260–340 g, and age-matched normotensive Wistar-Kyoto rats (WKY) weighing 280–360 g. Rats were delivered from the Centre for Experimental Medicine of the Medical University of Białystok (Poland). Animals had free access to food and water and were housed in plastic cages in a temperature-controlled room at 22 ± 1 °C under a 12/12 h light/dark cycle. Experimental protocols were approved by the local Animal Ethics Committee in Olsztyn (80/2017). In conscious SHR and WKY rats, systolic blood pressure (SBP) was recorded by the noninvasive tail-cuff method using the Rat Tail Blood Pressure Monitor (Hugo Sachs Elektronik, Harvard Apparatus; March-Hugstetten, Germany).

### 5.2. Chronic Treatment with Cannabidiol

(−)-Cannabidiol (CBD) 10 mg/kg or its vehicle was injected i.p. every 24 h for 14 days to the SHR and their normotensive controls WKY rats like it was described in [22]. The administration of the first CBD dose or its vehicle was preceded by systolic blood pressure (SBP) measurement in conscious rats. Twenty-four hours after the final dose, the SBP measurement was repeated. The systolic blood pressure (SBP) before the first dose of CBD (or its vehicle) was higher in SHR than in age-matched WKY rats (180 ± 10 mmHg, *n* = 7, vs. 110 ± 12 mmHg, *n* = 5, *p* < 0.001). Two-week administration of CBD or its vehicle did not modify SBP in any group as described previously (Table 1 in [22]). Left atria were collected 24 h after the last dose of CBD or its vehicle.

### 5.3. Isolated Atrial Preparations

Contractile function was studied as previously reported [22,29]. In brief, rats were anesthetized by i.p. injection of pentobarbital sodium (300 μmol/kg), and hearts were excised. Right and left atria from Wistar, SHR, and WKY rats were dissected and mounted in 10 mL glass chambers of the organ bath. Right atria worked spontaneously. Left atrial preparations were continuously electrically stimulated by using a bipolar platinum electrode with square wave pulses (just over the threshold, 5 ms duration, 2 Hz). Each preparation was stretched to ~5 mN. Force and frequency (rate) of contractions were recorded using an isometric force transducer (PIM 100RE, BIO-SYS-TECH, Białystok, Poland) and collected with DASYLab software (version 9.0, DasyTec, Waltham, MA, USA). Isolated atria were equilibrated in the organ bath for 30–60 min containing Tyrodesʼs solution (mM): NaCl 118, KCl 4.8, MgSO_4_ 1, NaHCO_3_ 29, NaH_2_PO_4_ × 12H_2_0 1, CaCl_2_ 2.25, glucose 10, Na-pyruvate 5, and EDTA 0.04 (pH 7.4; 37 °C) and gassed with carbogen (95% O_2_ and 5% CO_2_) under normoxia conditions.

### 5.4. Isolated Papillary Muscle

The papillary muscles were dissected from the right and left ventricles of Wistar rats, mounted in 10 mL glass chambers of the organ bath, and suspended vertically to isometric force transducers (FT20, Hugo Sachs Elektronik, March-Hugstetten, Germany) under 5 mN tension [38,43] and electrical-field stimulation (just over the threshold, 5 ms duration, 2.5 Hz). Data were collected by the data acquisition system (LabChart Pro, ADInstruments, Sydney, Australia). Isolated papillary muscles were equilibrated in the organ bath for 90 min containing Tyrodeʼs solution (mM): NaCl 119,8, KCl 5.45, MgCl_2_ 1.05, NaHCO_3_ 22.6, NaH_2_PO_4_ × H_2_0 0.42, CaCl_2_ × 2H_2_0 1.8, glucose 5.05, ascorbic acid 0.25, and EDTA 0.05 (pH 7.4; 37 °C) and gassed under normoxia conditions with carbogen (95% O_2_ and 5% CO_2_). Muscle force was normalized by cross-sectional area calculated from the length and weight of the muscle, assuming a muscle density of 1.06 g cm^−3^ [73].

### 5.5. Hypoxia/Reoxygenation Experimental Protocols

The hypoxia conditions were obtained in all cardiac preparations by replacement of carbogen gas to 95% N_2_ and 5% CO_2_ in the organ bath [74] for 30 min with following, 30 min of reoxygenation (H/R) [75]. As mentioned before, different protocols were used, and presented in Figure 1.

### 5.6. Drugs

(−)-Cannabidiol for chronic experiments (THC Pharm GmbH, Frankfurt, Germany); (−)-Cannabidiol for acute experiments (Tocris Bioscience, Bristol, UK); (−)-Isoprenaline (±)-bitartrate salt (Sigma-Aldrich, Munich, Germany); pentobarbital sodium (Biowet, Puławy, Poland); Tween 80 (Sigma-Aldrich, Munich, Germany). Stock solutions of isoprenaline were prepared using distilled water. For chronic experiments, rats were injected i.p. with CBD (10 mg/kg) every 24 h for 14 days or received CBD vehicle [ethanol, Tween 80 (Sigma-Aldrich, Munich, Germany), 0.9% NaCl—3:1:16; 1 mL/kg]. For acute experiments, CBD was dissolved in dimethyl sulfoxide (DMSO, Sigma-Aldrich, Steinheim, Germany). Further dilutions were created with Krebs solution. In that manner, the final concentration of DMSO in the tissue bath was <0.01%.

### 5.7. Statistical Analysis

Results are given as the mean ± SEM (*n* = number of animals). Maximal effects of isoprenaline (E_max_) and its potency [pEC_50_ values defined as the negative logarithm of the EC_50_, i.e., concentration exerting 50% of maximal effects (E_max_)] were evaluated from the particular concentration-response curves. In acute experiments, positive chronotropic effects in right atria are shown as changes from baseline values, and positive inotropic effects are shown as changes in mN (left atria) or mN/cm^2^ (papillary muscles) and as a percentage of the maximum responses of the control group to ISO. For chronic experiments, positive inotropic responses to ISO in left atria are expressed as changes in force (delta, mN) of contractions and as percentages of basal values. Intergroup statistical comparisons were performed by one-way analysis of variance (ANOVA) followed by Bonferroni′s multiple comparison tests for selected pairs of the entire data set or *t*-tests where appropriate. Differences were considered significant when *p* < 0.05. Statistical analysis was performed using Graph Pad Prism version 5.0 (La Jolla, CA, USA).

## 6. Conclusions

We demonstrated that hypoxia/reoxygenation modified the function of isolated spontaneously beating the right atria and paced left atria and papillary muscles from right and left ventricles in a manner dependent on cardiac region and β-adrenergic prestimulation. Hypertension accelerated left atrial recovery during reoxygenation in contrast to normotensive atria, despite similar declines in contraction force during hypoxia. Right atria are the most resistant to hypoxia, and their function was almost completely restored in normotensive controls, regardless of prior β-adrenergic stimulation. Conversely, the isoprenaline-induced chronotropic effect in the right atria was not affected by hypoxia/reoxygenation and acute CBD pretreatment. Hypoxia significantly decreased the force of contraction of both the left atrium and papillary muscles of the right and left ventricles, independently of the β-adrenergic prestimulation. However, previous β-adrenergic stimulation improved recovery in isolated cardiac tissues derived from low—(left atria and right ventricle) but not high-pressure chambers (left ventricle). As well, acute (but not chronic) CBD administration increased the effects of isoprenaline on left atria and papillary muscles isolated from the right ventricle, but not from the left ventricle. There are opposing hypotheses regarding the beneficial effects of both blockade of β_1_-adrenoceptors and activation of β_1_–, β_2_–, or β_3_–adrenoceptors against ischemia/reperfusion injury [33]. Our data tilt the scale of arguments in favor of those hypotheses to the rather poor contribution of β—adrenergic activation to protection against hypoxia-induced injury. Finally, it should be noted that in the whole heart, the beneficial effects of CBD prestimulation can be further amplified by its known anti-inflammatory and antioxidant properties as well as its ability to improve vascular function. The results of clinical trials conducted around the world suggest that cannabidiol will become more widely used in clinical practice (see Introduction). Our findings emphasize the necessity for further preclinical studies to investigate the cardioprotective potential of cannabidiol. However, these studies should be performed on animals with intact vascular systems.

## Figures and Tables

**Table 1:** Influence of cannabidiol (CBD) on isoprenaline (ISO)-induced increases in the rate of rat right atria and force of contractions of left atria and right and left ventricular papillary muscles, respectively.

	Control	CBD	H/R	CBD H/R		Control	CBD	H/R	CBD H/R
	Right Atrium					Left Atrium			
basal (bpm)	341 ± 16	346 ± 17	347 ± 8	343 ± 18	basal (mN)	2.4 ± 0.1	2.3 ± 0.1	2.2 ± 0.1	2.3 ± 0.1
30 min after CBD/vehicle (bpm)	359 ± 15	373 ± 12	323 ± 18	323 ± 14	30 min after CBD/vehicle (mN)	2.2 ± 0.1	2.2 ± 0.1	2.1 ± 0.1	2.1 ± 0.1
E_max_^1^	111 ± 16	90 ± 10	106 ± 17	95 ± 16	E_max_^2^	99 ± 1	146 ± 21 *	51 ± 10 ^#^	46 ± 8
pEC_50_	8.8 ± 0.2	8.5 ± 0.1	8.5 ± 0.2	8.7 ± 0.2	pEC_50_	8.3 ± 0.1	8.4 ± 0.2	8.2 ± 0.2	7.9 ± 0.2
*n*	10	10	10	10	*n*	10	10	10	10
Right Ventricular Papillary Muscles					Left Ventricular Papillary Muscles				
Basal (mN/cm^2^)	7.9 ± 0.2	7.9 ± 0.9	7.8 ± 0.9	8.7 ± 1.0	basal (mN/cm^2^)	23.0 ± 6.5	23.4 ± 4.4	20.0 ± 4.2	23.8 ± 5.4
30 min after CBD/vehicle (mN/cm^2^)	7.5 ± 0.5	8.0 ± 0.7	6.1 ± 0.6	6.3 ± 1.6	30 min after CBD/vehicle(mN/cm^2^)	25.0 ± 6.7	26.1 ± 4.0	18.0 ± 4.5	24.3 ± 6.1
E_max_^2^	98 ± 4	108 ± 29	56 ± 8 ^#^	56 ± 16	E_max_^2^	100 ± 25	105 ± 17	28 ± 6 ^##^	47 ± 8
pEC_50_	7.0 ± 0.1	7.6 ± 0.3	6.5 ± 0.2	7.1 ± 0.3	pEC_50_	6.8 ± 0.2	6.8 ± 0.2	7.3 ± 0.2	7.0 ± 0.3
*n*	6	6	7	9	*n*	7	6	10	9

Basal parameters were recorded before (the first line) and 30 min after (the second line) cannabidiol (CBD; 1 µM) or its vehicle administration. The concentration curves for isoprenaline were constructed before (control, CBD) or after hypoxia and reoxygenation (H/R, CBD H/R). For experimental protocols, see Figure 1. E_max_ for ISO was expressed as maximal changes from baseline ^1^ (right atria) and as percentages of the maximum responses to ISO ^2^ (left atria and papillary muscles) in the control group. Isoprenaline (0.01 nM–10 µM) was given cumulatively in experiments with atria and papillary muscles isolated from Wistar rats (*n* = 6–10). Data are given as the means ± SEM. * *p* < 0.05; significant effect of CBD; ^#^ *p* < 0.05, ^##^ *p* < 0.01 significant effect of H/R; one-way analysis of variance (ANOVA) with Bonferroni post hoc test; bpm, beats per minute.

**Table 2:** Influence of chronic administration of cannabidiol (CBD) on isoprenaline (ISO)-induced changes in the force of contractions of left atria isolated from spontaneously hypertensive rats (SHR) and normotensive Wistar–Kyoto rats (WKY).

	WKY	WKY CBD	SHR	SHR CBD
basal (mN)	2.4 ± 0.1	2.4 ± 0.1	2.4 ± 0.1	2.4 ± 0.1
E_max_^1^ (Δ, mN)	0.7 ± 0.1	0.8 ± 0.2	0.7 ± 0.1	0.6 ± 0.1
pEC_50_^1^	7.4 ± 0.2	7.6 ± 0.2	8.1 ± 0.2 ^#^	8.5 ± 0.2
E_max_^2^ (% of basal)	54.9 ± 11.1	68.7 ± 19.4	32.5 ± 6.0	27.1 ± 5.2
pEC_50_^2^	7.5 ± 0.2	7.6 ± 0.2	8.0 ± 0.2	8.5 ± 0.2
*n*	5	7	7	6

Cannabidiol (CBD) or its vehicle was given chronically (every 24 h for 14 days, i.p., 10 mg/kg). Isoprenaline (ISO) was administered cumulatively (0.01 nM–30 µM) to organ baths with left atria isolated from spontaneously hypertensive rats (SHR) and their corresponding control (Wistar–Kyoto rats, WKY) collected 24 h after the final dose of CBD or vehicle (for details, see Figure 1). Isoprenaline was administered after the previous hypoxia/reoxygenation (30/30 min). Data are expressed as changes (delta) in force of contractions ^1^ and as percentages of basal values ^2^ and given as the means ± SEM of 5–7 rats. ^#^ *p* < 0.05, SHR significantly different from WKY; one-way analysis of variance (ANOVA) with Bonferroni post hoc test.

## Data Availability

The datasets used and/or analyzed during the current study are available from the corresponding author upon reasonable request.
